# Large-Scale Estimation and Analysis of Web Users' Mood from Web Search Query and Mobile Sensor Data

**DOI:** 10.1089/big.2022.0211

**Published:** 2024-06-19

**Authors:** Wataru Sasaki, Satoki Hamanaka, Satoko Miyahara, Kota Tsubouchi, Jin Nakazawa, Tadashi Okoshi

**Affiliations:** ^1^ Graduate School of Media and Governance, Keio University, Fujisawa-shi, Japan.; ^2^ Yahoo Japan Corporation, Chiyoda-ku, Japan.; ^3^ Faculty of Environment and Information Studies, Keio University, Fujisawa-shi, Japan.

**Keywords:** mood estimation, web search, mobile sensing, COVID-19 analysis

## Abstract

The ability to estimate the current mood states of web users has considerable potential for realizing user-centric opportune services in pervasive computing. However, it is difficult to determine the data type used for such estimation and collect the ground truth of such mood states. Therefore, we built a model to estimate the mood states from search-query data in an easy-to-collect and non-invasive manner. Then, we built a model to estimate mood states from mobile sensor data as another estimation model and supplemented its output to the ground-truth label of the model estimated from search queries. This novel two-step model building contributed to boosting the performance of estimating the mood states of web users. Our system was also deployed in the commercial stack, and large-scale data analysis with >11 million users was conducted. We proposed a nationwide mood score, which bundles the mood values of users across the country. It shows the daily and weekly rhythm of people's moods and explains the ups and downs of moods during the COVID-19 pandemic, which is inversely synchronized to the number of new COVID-19 cases. It detects big news that simultaneously affects the mood states of many users, even under fine-grained time resolution, such as the order of hours. In addition, we identified a certain class of advertisements that indicated a clear tendency in the mood of the users who clicked such advertisements.

## Introduction

The ability to estimate the current positive/negative affective states (such as mood) of web users is useful not only for realizing user-centric services tailored to specific states of individual users and analyzing the current mood states of people at a larger level, such as the full web service, or people in different cities, regions, or with different demographic attributes.

However, it is difficult to determine the mood states of web users outside a controlled in-lab configuration, particularly in the real-world situation of commercial web services. The first problem is the difficulty in sensing modality selection. Typically, sensing and determining the emotional state of a person requires psycho-physiological data such as heart rate,^1^ heart rate variability, electrocardiogram, and electroencephalogram data.^2,3^

However, collecting such data in real-world conditions of web users is not feasible owing to the low penetration rate of such sensors in society, the additional burden on users to use such devices, and the lack of social acceptance for collecting such data. The second problem is the difficulty of collecting ground-truth labels on the user's mood states. User annotation methodologies, where users provide their subjective views on mood states, are widely used during the data-collection phase.

However, this approach is not always effective because the users might find it cumbersome to answer repeated questions and sometimes forget to answer the questionnaire. In commercial web services, it is not feasible to repeatedly send such questionnaires to users.

To solve the first problem, we built a search-query mood model (QMM), which estimates the user mood states from web search queries, an easy-to-collect and non-invasive modality, to explain the mood states of users. We focused on the fact that almost all Internet users regularly use search engines in their daily lives. Also, the fact that almost all web services store user search queries as logs for big data (BD) analysis means that the proposed method is easy to be deployed in a wide range of web services and also has the advantage of being able to estimate user status retroactively.

To solve the second problem, we built a sensor mood model (SMM) to estimate mood states from smartphone sensor data in advance. Then, for data augmentation purpose, the output of SMM is supplemented with ground-truth label data of the QMM. This allowed us to generate more label data when constructing QMM, resulting in a better-performing QMM with an 11% improvement in area under the curve (AUC).

**FIG. 1.**
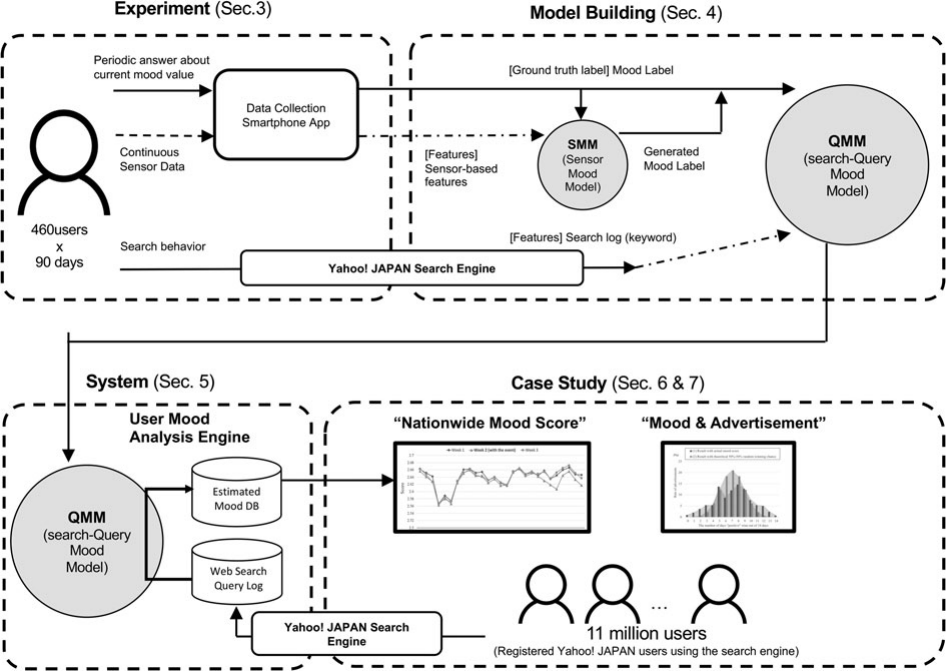
Roadmap of this study.

Figure 1 illustrates our study roadmap.

(1) We conducted a data-collection study with 460 participants for 90 days to collect their continuous sensor data from their smartphones (Experiment for Building Models section).
(2) Next, we built SMM, which estimates the participant's mood states of participants, both sensor data and the user annotation. We generated estimated mood states for all time frames during the data-collection study period by applying the built SMM, Then, by combining the web search logs of the 460 participants during the data-collection period and mood states (both the original annotation of users and SMM output), we trained QMM to estimate the mood of users from their search-query data.
(3) We deployed a user mood analysis system in our server-side stack. This system calculates mood scores of registered Yahoo! JAPAN users (>11,000,000 users in Japan) every 3 hours by processing their web search queries and inputting them into the built QMM model. The average of all user scores was calculated as the “nationwide mood score.”
(4) Our case studies confirmed that the proposed mood score successfully captured user mood changes in different time scales. First, the score showed the weekly mood rhythm of people (which dropped every first working day of the week and increased again every weekend). Second, longer-term tracing of the mood of the people during the COVID-19 pandemic in 2020 captured (inverse) synchronization against the daily number of new COVID-19 cases.

Significant differences in mood drop between 47 geographical regions with different numbers of cases were confirmed. Further, we also found that our mood score can successfully capture the change in people's moods influenced by significant news, which simultaneously affects the mood states of many users. We further analyzed the stored historical logs of 100 recent advertisement projects, including when and who viewed the advertisements and whether they clicked on them.

We identified a certain class of advertisements that indicated a clear tendency in the mood of the users who clicked such advertisements (Case Study: Mood and Advertisement section). Although it is important to solve ethical issues for the “actual” mood-based advertisement delivery, this finding is among the scientific contribution of this study.

Our study makes the following three-fold contribution.

- To boost performance in estimating user mood states from search-query data, we used a novel two-step mood classification with different models, namely SMM and QMM, to generate more mood status labels for QMM and improve the overall classification performance.
- We built a mood analysis system in our server stack where mood scores of registered Yahoo! users in Japan (>11 million users) are estimated every 3 hours based on machine-learning models. This is the first time a machine-learning-based system for estimating affective mood states from real-world data has been deployed and evaluated on a large scale and long term.
- We investigated the relationship between the nationwide mood score calculated from the search-query data of >11 million users and several use cases such as weekly rhythms, the number of new coronary cases, and big news. We also analyzed the impact on ad clicks as a use case in which the users were randomly selected.

## Related Work

Extensive studies on emotions began to be conducted in the 19th century, with a well-known study conducted by Darwin,^4^ who reported that emotion is a product of evolution and that emotions induce actions favorable to survival.^5^ Numerous emotional modalities and their respective physiological responses have been studied.^6,7^ Emotional states are known to affect cognitive and athletic abilities, and they are reported to affect both human–human and human–machine interactions.

Picard generalized this research field as affective computing.^8^ Many studies and systems have been proposed to detect and utilize users' emotions^9–13^ in this field. Several methods to determine user emotion have been proposed, focusing on physical characteristics^14,15^ and text data.^16,17^ Our study focuses on the mood states of users. The mood is different from emotion in several ways,^18^ including the tendency for the mood to last longer than emotion and that it is usually a cumulative reaction. In contrast, emotion is a more spontaneous reaction.

Mood estimating research on smartphones has been conducted in recent years. The advantages of this approach are the sensing capability of smartphones using a wide variety of embedded sensors, which reduces the burden on users of wearing various dedicated sensors; the easiness to use these devices, and the high penetration rate of smartphones (>95% in Japan).

Most studies have constructed a classification model that determines moods from the user's contextual data obtained from smartphone sensor data and self-reporting annotation (mainly via experience sampling methods).^19^ MoodScope^12^ investigated the effects of user context on a user's mood based on smartphone sensor data. In this study, we also built a model to estimate mood states from smartphone sensors by taking advantage of the smartphone's ability to run multiple sensors in the background. This will help generate ground-truth data that would be labeled data for the model to estimate the mood from search queries.

Focusing on the data on the web, researchers are continuously working on estimating users' emotional states by analyzing text from the users. Some studies estimate user emotional states from search queries on the web search engine, relying on emotion-related keywords (e.g., adjectives from which emotion has been easily inferred).^20^ However, in real-world situations of the web search engine, it is difficult to estimate since most search queries have only a few words (1.9 according to Tsubouchi et al.^21^) and mainly nouns.

More recently, analysis of text data on social networks such as Twitter^17,22^ and Facebook^23,24^ has been actively conducted. This type of text data is considered to contain more words and longer sentences, possibly including words related to their emotional states. In addition, under the influence of the COVID-19 pandemic, research on estimating depression mood from social media has been actively conducted in recent years.^25,26^

However, user data on such major social networks are not always accessible from the viewpoint of individual web services unless users link their accounts to their social media accounts and permit the web service to access and analyze their updates. Contrastingly, our approach relies on queries to the search function, which is commonly deployed on each website, and thus is easier to be introduced by each web service. Although the novelty of our method lies in the combined use of different types of mood estimation models from smartphone sensors and search queries, once a two-step QMM is constructed, the mood states can be estimated with search-query data without smartphone data after that.

Multimodal (MM) affect detection systems have gradually been reported in recent years.^27^ While unimodal (UM) detection involves the using single modality (e.g., facial features, gestures), MM systems fuse two or more modalities for affect detection. MM systems are consistently more accurate than UM systems, with a mean improvement of 9.83% (median 6.60%), according to the meta-analysis.^28^ One of the keys is the effective fusion of multiple modalities.

There are three ways to fuse signals from different modalities: Data Fusion, Feature Fusion, and Decision Fusion.^29^ Data Fusion is performed on raw data for each signal and can only be applied when the signals have the exact temporal resolution. Feature Fusion is performed on the set of features extracted from each signal. The SMM is an example of Feature Fusion since the model is trained by merging features from multiple smartphone sensors.

Decision Fusion is performed by merging the classifier's outputs for each signal. The method proposed in this study, in which the SMM output is complemented with the QMM label data, is classified as this fusion method. Although Decision Fusion is the most commonly used approach for MM Human Computer Interaction, MM detection combining smartphone sensors and search keywords has not been conducted earlier, and this study is the first attempt.

There is a technique called “federal learning”^30,31^ that combines multiple machine-learning models in a step-by-step manner. This is a method in which data-generating models learned locally on the client are aggregated on the server to learn the entire model. The problem is that the recent acceleration of BD has increased the learning cost due to the enormous amount of data in the cloud. Federal learning can be used to address this issue, which is suitable for a learning environment where data are distributed rather than aggregated in the cloud.

In this study, considering the learning cost on the client, we did not train the models on the client but aggregated the client data to the server and trained the machine-learning models step-by-step on the server. However, the latest research has proposed more extended architectures for federal learning,^32^ and we will discuss their potential applications in the Discussion section.

## Experiment for Building Models

We first conducted a data-collection study with 460 users for 90 days (from November to December 2019). We collected continuous data from various types of smartphone sensors as well as the user's subjective mood evaluation (up to six times a day) as the ground-truth label. We also obtained the search query data during the experiment associated with the participants' ID from our server stack, where the search logs of all users are stored.

### Participants

Participants were recruited through an external agency. The recruitment criteria were as follows: (1) the age should be in the range of 18–59 years; (2) must own an active Yahoo! Japan registered account; (3) must use Yahoo! Japan search functionality once or more times per week and should have performed a search at least once in the last month; (4) must own and use an Apple iOS smartphone as a private primary phone in daily life; and (5) must be using an iPhone 7 or later and iOS version 12 or above. During recruitment, the participants were informed that this study was “an experiment about your condition.”

We successfully recruited 460 participants. Finally, we used data from 338 users, excluding those who stopped data collection during the experiment or who could not collect enough data. The participants were from different areas of Japan (geographically distributed), consisting of 121 men and 217 women, aged between 19 and 54 years (average: 36.92), and with various occupations (civil servants: 4.4%, business executives: 0.9%, company employees (clerical: 22.2%, technical: 8.0%, other: 16.6%), self-employed: 3.6%, freelancers: 0.9%, homemakers: 28.7%, part-time job employees: 9.2%, students: 1.8%, unemployed: 1.8%, others: 2.1%).

### Experimental setup

**FIG. 2.**
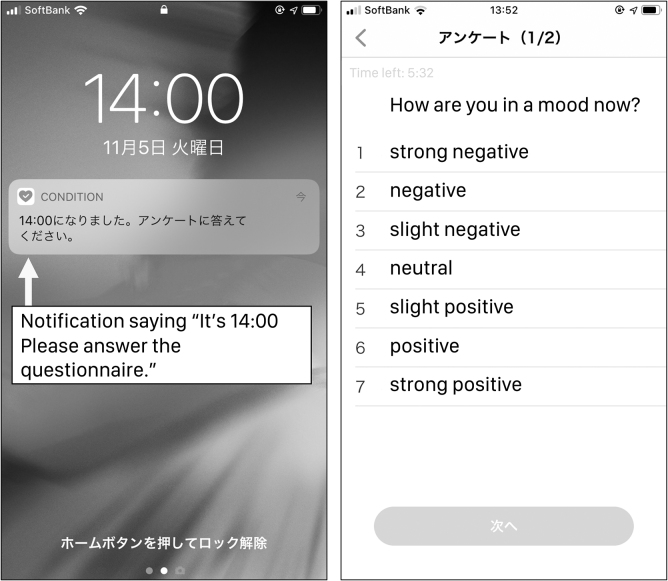
Screenshot of data collection application.

We developed a dedicated smartphone application, as illustrated in Figure 2. The application was developed for the iOS platform for several reasons. First, the market share of iOS is bigger than that of Android in the Japanese market; thus, recruiting participants was easier. Second, the number of Apple iPhone models (e.g., iPhone 7, 7Plus, 8, 8Plus, X, and 11) is smaller than that of Android phones (hundreds of models by dozens of manufacturers with different OS-level optimization in power management, sensing, etc.).

Thus, we could easily test the application with such phone models to achieve higher execution stability. Third, thanks to the iOS AWARE Framework,^33,34^ we can implement and deploy an application that can continuously collect various sensor data despite iOS being a rather strict environment as a sensing platform than Android.

Once the application was installed on the smartphone of a user, it continuously collected multiple types of data from the embedded sensors of the phone, as detailed in Table 1, and periodically uploaded the data to the server.

**Table 1.** Sensor type and sensing frequency

***Sensor type***	***Sensing frequency***
Accelerometer	10 Hz
Barometer	1 Hz
Battery status	1 Hz
Gyroscope	10 Hz
Location	1/180 Hz
Network type	(changed event-based)
Weather (from OpenWeather)	1/60 Hz
Screen status (on/off)	(changed event-based)

The application was also able to issue notifications (as shown on the left-hand side of Fig. 2) at intervals configured by the developer to initiate data collection of experience sampling method style data. The delivery timing of such notifications to the smartphone devices differed for each user owing to the application's real-world random behavior on each device and various network-related variables.

This behavior generates some randomness in the timing to reduce possible bias in the experiment. Once the user responded to the notification, the application opened a questionnaire on which the users reported their current mood states on a seven-level Likert scale (1. strongly negative; 2. Negative; 3. moderately negative; 4. Neutral; 5. moderately positive; 6. positive; and 7. strongly positive).

### Experimental procedure

Our experimental procedure consisted of the following three steps:

(1) Each participant had a telephonic meeting with a study researcher at the beginning of the study and received instructions, including an overview, purpose, collecting data such as type of mobile sensor and search query, schedule, and tasks about the study, which was followed by the signing of a consent form. Because the experiment involved the participants' sensitive information and data, including sensor data, subjective mood states, and search-query data, we carefully protected the participants' ethical-related rights by carefully communicating with them.

(2) The participants installed and launched our software on their smartphones. They granted the following permissions to the application: push notification, motion and fitness activity, and location (configured as “always”) data sensing feature of the iOS platform.

(3) After the initial meeting, the 90-day study period started. During this period, a push notification appeared six times daily. (at ∼8:00 AM, 10:00 AM, noon, 2:00 PM, 4:00 PM, and 6:00 PM, but the actual notification timings of the client varied by several minutes.) Each participant was asked to proceed with the survey within 2 hours after the delivery of the notification. When the participants opened a notification, the application screen (Fig. 2) appeared, and they were asked about their mood on a seven-level Likert scale. The participants selected their states, and after a confirmation prompt, the answer was submitted to the server.

### Reward

We created an instant point reward system for the application. Each participant scored 0, 20, 30, or 40 points for 0–3, 4, 5, or 6 answers, respectively, daily, and the reward points accumulated throughout the study period. When a participant reached the configured minimum total reward points, that is, 1500 points (by answering four answers every day for 75 days or six answers every day for 38 days), they received a payment of 3000 yen. They received an additional payment of 2000 yen when they exceeded 2000 points.

## Two-Step Model Building

As introduced in Figure 1, we propose our novel method of two-step model building. Based on the obtained data from the experiment in the previous section, we built two different machine-learning models, “search-Query Mood Model (QMM)” and “Sensor Mood Model (SMM).”

To build the models, we used a widely used approach in the activity recognition research area^35^ to build a classifier with time-frame-based feature extraction from time-series sensor data. For our models, the frame length was 3 hours. (The detailed model-building methodologies and their performance evaluation are provided in the Supplementary Material.)

### Overview

**FIG. 3.**
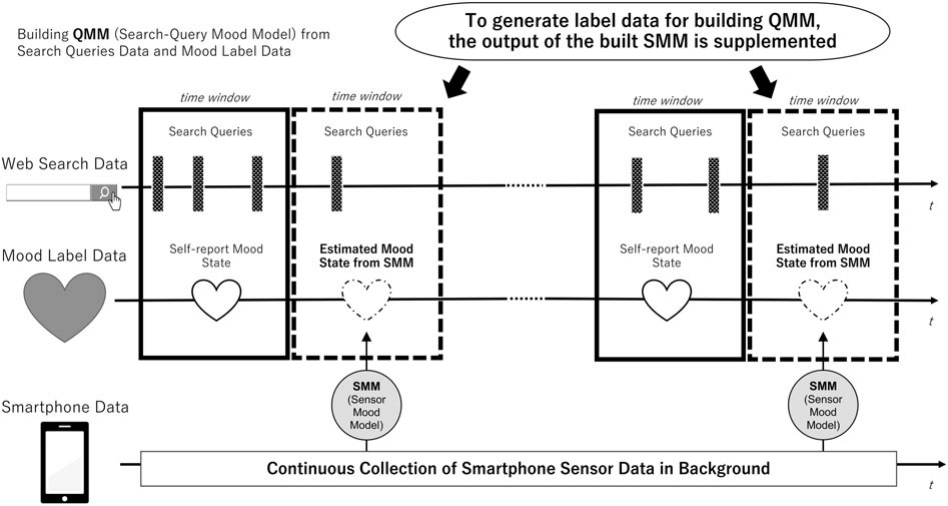
Overview of proposed two-step model building.

Figure 3 shows the concepts of our approach for building the QMM with the supplement of the SMM's output as the ground-truth label data. We present an overview of those models and their combined use for improving performance. Mood labels and search query data were collected to build the QMM that estimates mood states from search queries. However, there were not always mood labels in the time window (3 hours). Therefore, we constructed the SMM from smartphone sensors and mood labels.

Then, by supplementing the SMM's estimated mood values as mood labels to be used during QMM construction, the number of pairs of search query data and mood label data in the time window that could be used for training of QMM construction was increased, thereby improving QMM performance.

### Sensor mood model

SMM is a model that estimates the user mood score in a specific time frame (3 hours) from a corresponding set of features extracted from the smartphone sensor data.

For each user and 3-hour frame, a pair of the calculated features and the user's self-reported mood states (as the ground truth label, if it is available) were combined and input to the ML model as training data. We built a single SMM from all the available training data obtained from all users in the data collection. After data pre-processing, our SMM resulted in 0.721 in micro-*F*1 score and 0.597 in macro-*F*1 score.

### Query mood model

QMM is a model that examines the relationship between the web search query and the user's mood score during the search behavior. For each user, QMM was trained as a logistic regression model for each 3-hour frame from the data of the user mood score and search behavior during the frame. The training was performed using the following regression equation:


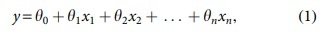


where *y* is the mood score, and 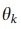 is the learned weight. *x_k_* is the search query assigned to a feature, and it has a value of 1 if it was searched in that frame, and 0 if it was not. *x_k_* indicates only whether the query is searched, and it does not indicate the number of searches. For multi-search words, we extracted each word and counted one for each according to the rule of *x_k_*.

### Combinational use with QMM and SMM

During the training of QMM, some frames missed the mood score mainly because the user's raw answer to the mood questionnaire was unavailable, although both the search behavior and mood score were required. To handle such situations, using pre-trained SMM and collected sensor data is an effective means of generating mood scores for the training.

Contrastingly, the mood score estimated by SMM was prepared constantly since our application continuously collected smartphone sensor data throughout the study period (except for some irregular cases where the application was not working). Therefore, SMM was used to supplement missing mood scores, creating labels for all periods during the data-collection study and increasing the training data for QMM.

We built two QMM models: one trained only from the questionnaire answer data (QMM without SMM) and the other with additional training data based on the SMM output (QMM with SMM). The cross-validation results are shown in Table 2, confirming SMM's effectiveness. (Note that the test set for QMM without SMM does not contain SMM-generated mood labels.) Compared with our baseline, we confirmed that the AUC increased from 0.583 to 0.623 (11%) in the case with additional data from SMM.

**Table 2.** Performance of search-query mood model

	***No. of label data***	***AUC***
QMM (with SMM (proposed))	**26,481**	**0.623**
QMM (without SMM)	13,301	0.583

Bold values signifies the better values.

AUC, area under the curve; QMM, query mood model; SMM, sensor mood model.

The table also shows that the amount of training data more than doubled with SMM, indicating that the more than doubling of the dataset used for training by SMM contributed significantly to the improvement in prediction accuracy. Consequently, we adopted a QMM trained with SMM and proceeded to our evaluation experiments.

Regarding the computational complexity, our proposed scenario increases the computational training complexity due to combining two different models because it supplements the outputs from the SMM where the QMM's label data are missing. The computational increase in the proposed scenario compared with the baseline (using QMM [without SMM] only) depends mainly on the behavior of supplementing the mood labels by the SMM.

This is because the SMM construction and the creation of its complementary labels affect the execution time and computational resources more than the increase in training data when building the QMM. The suppression of these computational increments will be discussed in the Discussion section.

## System Deployment

**FIG. 4.**
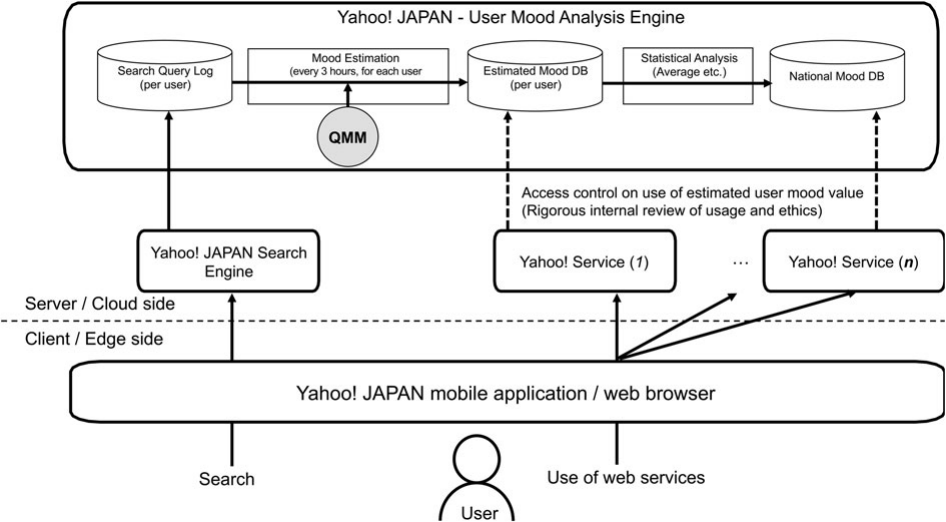
Yahoo! Japan user mood analysis engine.

Using the built QMM, we designed, implemented, and deployed a user mood analysis engine in our commercial server stack, as shown in Figure 4. The search-query log of all users was stored in the engine through the Yahoo! Japan Search engine, which is one of the most common search engines in the Japanese market with 80 million registered users. (Note that the population of Japan is about 126 million).

Using QMM, as explained in the Two-step Model Building section, the mood estimation module calculates the mood states of individual users from their search logs. The module can calculate the users' past mood states from the historical query log and their latest mood states every 3 hours. The system consists of a Hadoop cluster with 155 mappers and 200 reducers. One calculation took ∼10 to 40 minutes, depending on the current load of the Hadoop cluster (shared with other projects in our corporation) and the number of search queries.

Next, the statistical analysis module calculates the statistical values for use in analysis or application to other services. One calculated value was the nationwide mood score, the average of all users' mood states at a given time (the details are explained in the Nationwide Mood Score section). Yahoo! Japan has >70 Yahoo! Japan-branded services such as “search,” “news,” “shopping,” “auction,” “movie,” and “weather.” We believe that combining these various services with the estimated mood values allows for more sophisticated services for users.

For example, a service that hides shocking news for depressed users or a movie recommendation service that is more adaptive to the user's mood can be constructed. Such calculated values were used to determine the estimated mood data from other Yahoo! services, access to which is strictly controlled. Access controls on the use of the estimated mood state of a user were only granted after a rigorous internal review of the use and ethics-related issues.

## Case Study: Nationwide Mood

### Overview

It is difficult to find a baseline for evaluating the mood score we calculated, because mood is an affective state that humans cannot visualize and because no studies have calculated affective states for web data on a large scale.

To examine the value of calculating the mood scores of a large number of registered users, we assessed changes in mood scores across several different timescales and how they relate to social phenomena. All data analysis was conducted on Yahoo! Japan's internal servers after anonymization so that the research members could view only the statistical information.

#### Nationwide mood score

The index score we used for this examination was the nationwide mood score, the average mood score of all target users (∼11,000,000 users all over Japan). Since we use users' historical search query logs, the population from which the score is calculated comprises Yahoo! Japan's registered users who used our search engine at least once for each particular day or time. (Thus, the exact number of target users changes continuously.) Note that theoretically, the mood score as a calculated output from trained QMM and user search behavior value had no upper or lower limit because the mood score was not sigmoid fitted.

As a reasonable approximation, according to the corporate data of Yahoo! Japan (open to the public for advertisement sales), the demographics of the users of Yahoo! Japan geographically cover all prefectures of Japan, with a mixture of male (48%) and female (52%), and covering all age groups (male: 10s: 2%, 20s: 16%, 30s: 17%, 40s: 22%, 50s: 18%, 60s and above: 25%, female: 10s: 1%, 20s: 16%, 30s: 17%, 40s: 22%, 50s: 18%, 60s and above: 25%).

Since there is little deviation between the user attributes of the dataset on which the QMM was built and the target user attributes in the nationwide mood score, we applied the QMM to data with ∼11 million people. It is one of the attractive features of machine learning to apply models to large datasets.

By calculating and averaging the mood scores of all those users on a given day (or hour), we derived the mood value referred to as the nationwide mood score for Japan, daily (or hourly).

#### Comparative method

As described earlier, one of the challenges in this study is to show the effectiveness of the SMM output in the training of QMM. Thus, in this evaluation, we compared the QMM trained with the SMM output as our proposed method and the QMM without SMM as a comparative method. Note that all the conditions (algorithm, hyperparameters, and split ratio between the data) were the same between these two methods.

### Result 1: weekly and daily mood rhythms

The first case is an analysis of the daily mood score trend for 4 weeks. We intended to observe how the nationwide mood score changes within a month, which is relatively short term. For this analysis, we used a dataset from July 1, 2019, to July 28, 2019. We deliberately chose this period to exclude major breaking news stories (such as a significant shocking incident) that could influence the mood states of many users.

**FIG. 5.**
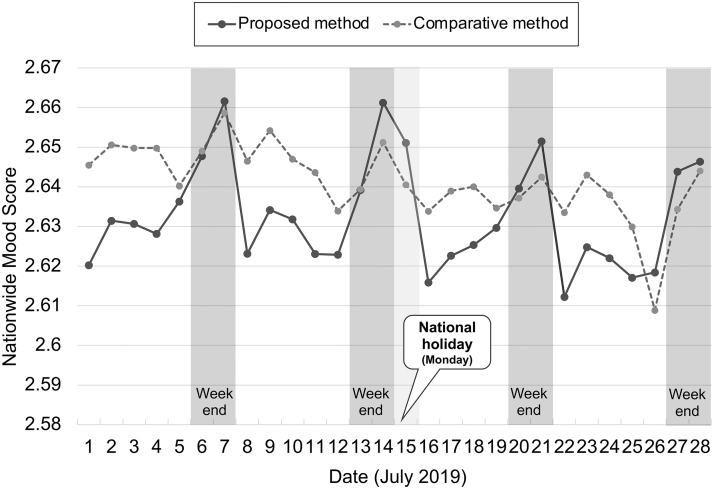
Weekly rhythm of daily nationwide mood score.

Figure 5 shows the resulting nation-wide mood scores for this period. The *x*-axis represents the date, whereas the *y*-axis represents the daily score. A very interesting result in the figure is that, with our proposed method, the scores are clearly positive on weekends and more negative on Mondays when the workday begins (or Tuesdays when Monday is a holiday on July 15).

Table 3 shows the statistics on all days throughout the year. We omitted the national holidays and counted the number of days whose daily score was equal to or higher than the previous day's score. Surprisingly, on all (46 out of 46) Mondays, the score decreased from the previous Sunday. Then, getting close to the weekend, especially on Fridays, Saturdays, and Sundays, the scores increased from the corresponding previous day.

**Table 3.** Statistics on weekly rhythm

***NationalMoodScore***	***Monday***	***Tuesday***	***Wednesday***	***Thursday***	***Friday***	***Saturday***	***Sunday***
Today ≥Yesterday	0.0%	77.1%	56.0%	44.2%	74.0%	94.0%	94.2%
Today <Yesterday	100.0%	22.9%	44.0%	55.8%	26.0%	6.0%	5.8%

#### Discussion

Although no ground-truth data exist on a nationwide mood, this tendency to feel better later in the week and then worsen again on Monday is considered an intuitive result in a society where several people work from Monday through Friday. This is called “Blue Monday.” According to a white paper released by the Ministry of Health, Labor, and Welfare,^36^ Monday is the day with the highest number of suicides in Japan. Another survey of 400 men and women found that most respondents in all age groups felt most depressed on Mondays.^37^

The proposed method seems to successfully express the rhythm of the mood change over the weekdays and weekends in an instructive manner, whereas such rhythm is unclear in the comparative method. From this result, we conclude that the proposed method better explains the weekly mood rhythm than the comparative method.

### Result 2: COVID-19 and people's mood

Our second evaluation aims at revealing how the mood score changed during the COVID-19 pandemic situation.

#### Pandemic waves and nationwide mood

First, we investigated the waves of the pandemic and mood scores in the nationwide granularity. We chose the mood score for Sundays because every Sunday is a holiday; otherwise, it was assumed that it would be difficult to discuss the analysis results owing to occasional holidays.

**FIG. 6.**
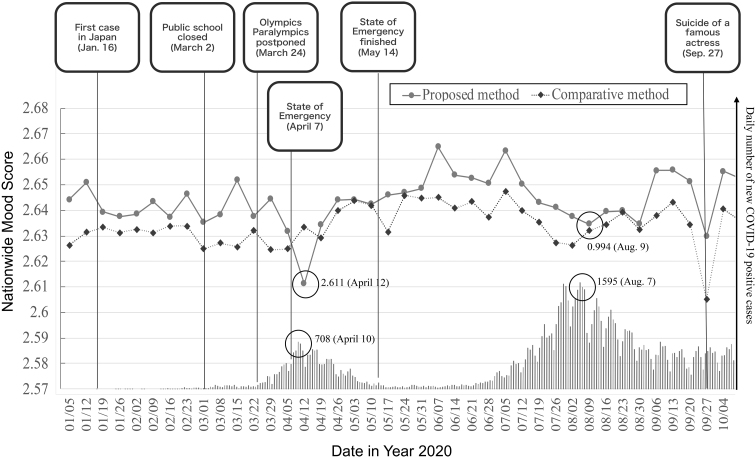
Nation-wide mood score and number of COVID-19 patients in year 2020.

Figure 6 shows the daily number of new COVID-19 cases and the change in the nationwide mood score. The value in the *y*-axis (left side, lines) indicates the scores calculated by the proposed and comparative methods. Also, the bars show the daily number of new COVID-19 cases in Japan.^38^

#### Causality analysis using convergent cross-mapping

As explained earlier, the possibility of a time-series relationship between the mood score and the number of COVID-19 infections was suggested. To demonstrate the relationship, we evaluated causality in time-series data of two variables using the convergent cross-mapping (CCM)^39^ analysis method. There are some conventional methods for evaluating causality in time-series data between two variables, such as Transfer-Entropy^40^ and Granger-Causality.^41^ However, since both methods assume that the target data have stationarity with stochastic processes, applying them to situation-dependent data is not appropriate.

To deal with such cases, Taken's Theorem^42^ is used to reconstruct the dynamical system's attractor followed by the data variables using past points. CCM reconstructs the state space using the optimal embedding dimension calculated by incorporating Taken's Theory and determines whether there is a causal relationship between the data if the prediction accuracy converges as the number of data points increases.

The overview of the CCM analysis procedure includes the following three steps.

**FIG. 7.**
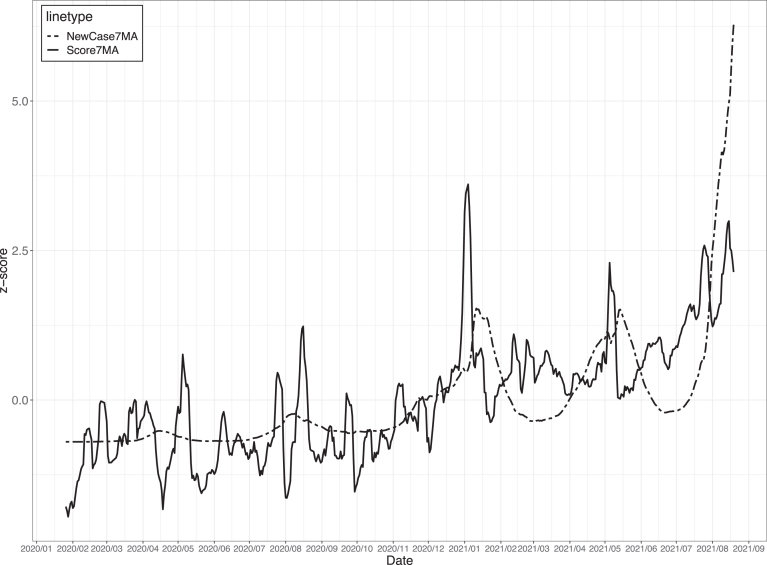
Standardization of nation-wide mood score (Score7MA) and the number of new COVID-19 cases (NewCase7MA) using 7-day moving average.

**FIG. 8.**
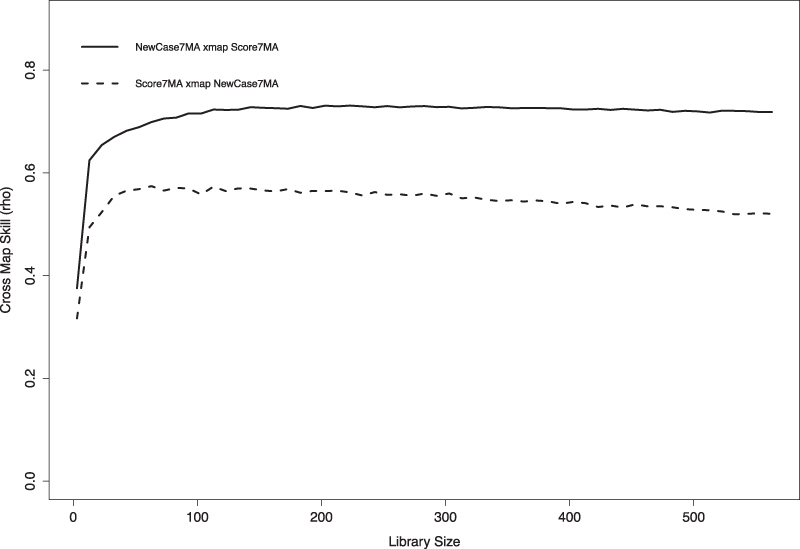
Causality identification result between Score7MA and NewCase7MA.

1. Data smoothing to remove noise data.
2. Check the embedding dimension using simplex projection^43,44^ and check the non-linearity using s_map.^45^
3. Analyze the data by applying CCM.

(Step1) Figure 7 shows the number of new COVID-19 cases and nationwide mood scores from January 2020 to August 2021, which were smoothed by applying a low-pass filter with a 7-day moving average. We further normalized both data so that the mean was 0 and the standard deviation was 1.

(Step2) By conducting a simplex projection with Root Mean Squared Error as the evaluation criterion to determine the optimal embedding, we varied the number of dimensions E from 1 to 24 (the square number of data points.^46^ We found that the prediction accuracy was lowest when *E* = 2 for both data. We also observed non-linearity for both data using s_map, which can be considered situation dependent.

(Step3) We conducted CCM with *E* = 2, as calculated in Step 2, and evaluated the results using correlation coefficients. Figure 8 shows the causality result by CCM, where the *x*-axis is library size, which means the time-series length used to construct the attractor, and the *y*-axis is the correlation coefficient.

The notation of the label in this figure is based on the original paper in which CCM was proposed,^39^ and it should be noted that “NewCasw7MA xmap Score7MA” represents the cross maps skills, which means how much the 7-day moving average of nationwide mood scores affects the 7-day moving average of the number of new COVID-19 cases. Through evaluation using CCM for the entire duration in Figure 7, we can confirm a causal relationship in both directions between the nationwide mood scores and the number of new COVID-19 cases.

Particularly, there is a strong causal relationship that nationwide mood scores affect the number of new COVID-19 cases, and the correlation coefficient increased as the library size increased with early convergence occurring.

There were five waves of COVID-19 cases in the period displayed in Figure 7 (first: March 2020–May 2020, second: July 2020–September 2020, third: October 2020–March 2021, fourth: April 2021–June 2021, fifth: July 2021–September 2021). Although the previous analysis for the overall period indicated causal relationships, a similar analysis using CCM for each COVID-19 wave showed no statistical relationship between the mood score and the number of new infections. (Some analyses could not be conducted since the data were confounded as linear and non-linear.) This suggests that the relationship can be captured by analyzing the data not within the short-term time window, such as COVID-19 waves, but from the perspective of a longer-term time window.

#### Differences in mood drops in different regions

Next, we decided to examine the relationship between the drop in the mood score and the spread of the pandemic at a geographically finer level. During the first wave of the pandemic (to April 12, 2020), most cases were identified in big cities with major airports with international flight routes, such as Tokyo and Osaka, including their surrounding areas, such as Kanagawa, Chiba, and Saitama. Moreover, other relatively rural areas away from the metropolitan areas generated no or minimal positive cases. This difference might have affected the change in people's moods.

We hypothesized that when mood scores were calculated by prefecture, the more COVID-19 cases a prefecture had, the more severe the drop in mood toward the peak of the first wave was observed. Thus, we divided the same data from about 11 million users into the 47 prefectures in Japan, according to user residential address data (used for the payment of several Yahoo! Japan paid services) in their account profiles, and then calculated the daily “Regional” mood scores for each prefecture.

**FIG. 9.**
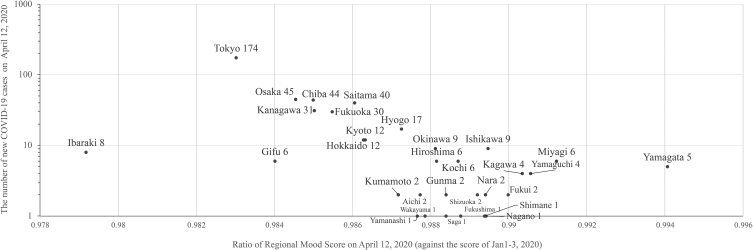
COVID-19 cases at the peak of the first pandemic wave and drop in people's mood in each prefecture.

Figure 9 shows the result of our analysis. The *y*-axis indicates the number of new COVID-19 cases on April 12, 2020 (the peak Sunday of the first wave). Meanwhile, the *x*-axis shows the relative value of the mood scores in each prefecture on that day, compared with the average scores of the same region during the new year holidays (January 1 to 3, 2020). The lower this number (to the left), the more severe drop in mood toward the peak of the first wave is observed.

A distribution from the lower right to the upper left is observed, indicating that the more COVID-19-positive cases prefectures had, the more severe the decline in mood. We conducted the correlation analysis and found the Pearson correlation coefficient as −0.43, indicating a moderate negative correlation.

#### Discussion

Regarding these analyses, the most remarkable point regarding our model (with the proposed model) is that the period of time when we collected sensor data and search queries for the model building was before the COVID-19 pandemic (from October to December 2019), as mentioned in the Experiment for Building Models section. This means that there is no possibility that search queries such as “COVID-19” could have been included in the trained QMM as features for negative mood values.

Again, there is no ground truth on the nationwide mood. However, because we can, nevertheless, observe the score trends of inverse synchrony with the number of COVID-19 cases and negative correlation with COVID-19 cases, we conclude that the nationwide mood score (with the proposed method) matches our intuition more.

### Result 3: big news that affects the mood of many users at once

While analyzing the nationwide mood scores shown in Figure 6, we also found that the score can successfully capture the change in people's mood influenced by the relay of big breaking news.

Figure 6 shows a sharp drop in the score on September 27, 2020. After some investigation, we realized that it was the day when the suicide of a famous Japanese actress was reported. From our investigation, at 8:29 AM on that day, the first tweet on Twitter^47^ reported that a breaking news text appeared on TV. (In Japan, big news stories are reported on a TV broadcast in the form of overlay texts on the TV screen.) Almost simultaneously, at 8:30 AM, the earliest breaking news article on her suicide was published on a web news site.^48^

**FIG. 10.**
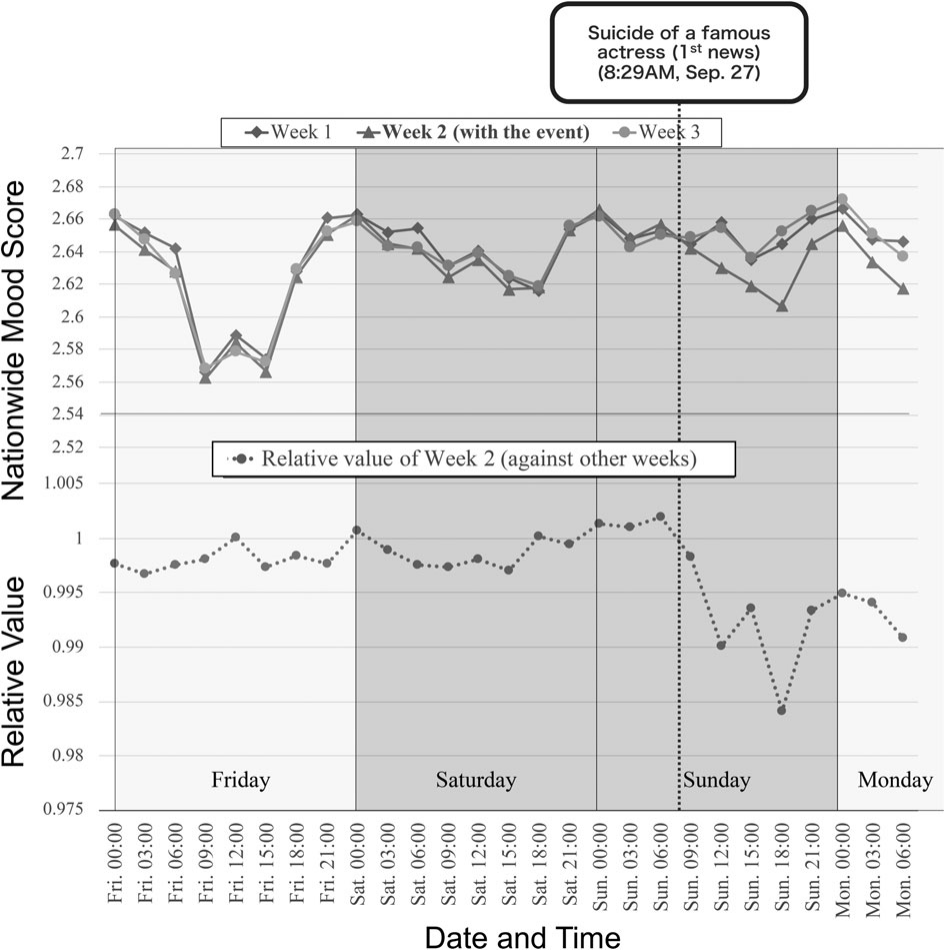
Score gap on the weekend of an actress's death.

We observed the trace of the nationwide mood score on that day by comparing it with similar traces on other weekends. Figure 10 (top) overlays the traces of the nationwide mood score for three different weekends, week 1 (the previous weekend), week 2 (the target weekend), and week 3 (the next weekend). When we look at the dotted line (which illustrates 8:30 AM on Sunday), the score of week 2 starts to decrease compared with the other 2 weeks.

Figure 10 (bottom) shows a trace of the relative score (the score of week 2 divided by the average of the week 1 and 3 values). We can clearly observe that the mood became negative after 8:30 AM on September 27, 2020, when the news was reported. From these results, we concluded that the nationwide mood score could detect large events that may affect several users' mood states, even in a fine-grained time resolution, such as the order of hours.

## Case Study: Mood and Advertisement

To investigate how web users' behavior correlates with their mood, we focused on web advertisements. This is because advertisement and user behavior (clicking) are common interactions among >70 Yahoo! JAPAN services.

We conducted an experiment on 100 advertisements (ads) cases that were delivered to our users in the past. We examined the impression (the number of times the advertisement was exposed to users) and click logs for those 100 ads and tested offline to find out whether there were ads that made a difference in whether users clicked or not, depending on their mood state just before their click.

### Dataset

The targeted dataset consisted of 100 ads served through Yahoo! JAPAN ad service. To ensure sufficient impression and click volume for the data analysis, we selected the 100 ads from historical and business data stored in our servers as the most recent 100 projects with a delivery record of at least 14 days. The targeted ads had at least ∼5 billion user impressions (views) and ∼3 million clicks during the 14-day period. User IDs and timestamps for impressions and clicks on ads are, respectively, stored in our server's internal storage.

Therefore, through the user ID in each impression or click log, we could link the user's history of organic search behavior. Through this link, the three-hourly mood score for each user could be calculated using our QMM model. Finally, the results of this calculation were used to analyze whether or not a user in a certain mood state clicked on an ad when they viewed it.

### Metrics

A pairwise method was used to investigate whether any ads changed the click state of an ad depending on the user's mood score. First, we organized the logs for the days the ads were served and converted the data into timestamp format, the user ID of the viewer, and whether it clicked/or not. Then, for each day, we randomly selected two records from all the records. If only one of the two extracted records was a “clicked log,” we compared the mood scores of the two records.

We marked “positive” when the mood score of the clicked user was higher and vice versa. This pairwise extraction process was performed 100 million times for each day of the log since we attempted several different numbers and confirmed that the variability was sufficiently small with this number of iterations.

### Result

#### Existence of mood-ad click correlation

**FIG. 11.**
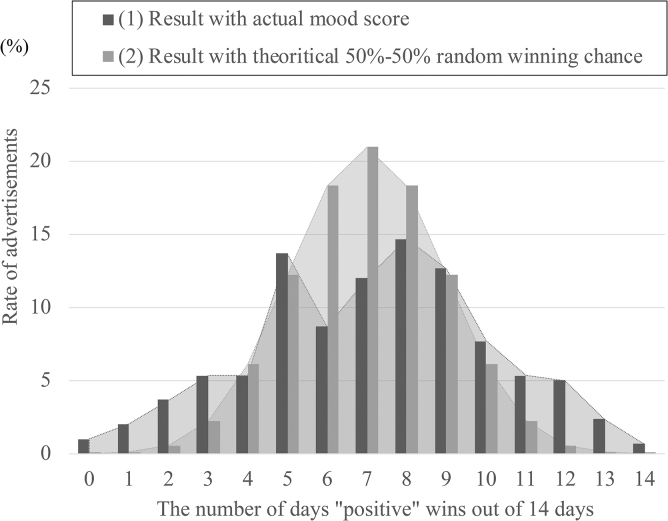
Distribution of advertisements by positive/negative ratio.

Figure 11 shows the results. On the *x*-axis is the number of days “positive” wins out of 14 days, whereas on the *y*-axis is the percentage of such ads (among 100 ads). Two lines are depicted in the figure; they are (1) the pairwise comparison result based on the actual estimated mood scores and (2) a theoretical line that would be drawn if we assume that the winner in each pairwise comparison attempt was completely random.

First, the shapes of the graphs (2) of random theoretical values have a convex shape that expands toward the center. Moreover, Line (2) is the result of a hypothetical experiment of 14 consecutive coin tosses. Thus, 14 consecutive wins (or losses) out of 14 tries are rare. (The probability of a positive winning in all 14 days is ∼0.01% when we randomly assign a score. This is the probability of 1 out of every 10,000 ad servings.)

Similarly, the same kind, probably with 13 wins (or losses), is ∼0.1% (1 out of 1000). However, a significant difference can be observed when looking at the actual data (1). The number of ads where the positive wins on all 14 days or losses on all 14 days could be found in ∼1% of the ads (100 times the theoretical result) if they were served by the mood score. Further, the results with “more than 13 wins or losses” scored 6 (6%) (60 times the theoretical result). We also observed that the shape of the convexity of Line (1) is spread out to look like it has been crushed on the side. From these results, we can confirm that there are, indeed, certain advertisements that are effective for delivery based on the mood scores of the recipients.

#### Detailed ad content analysis

First, we further examined in detail the ads that were more likely to be clicked on in the same emotional state for >13 out of 14 days. Although we cannot provide examples of actual advertisements delivered due to the confidentiality of the business-related information, we found that the ads that were most likely to be clicked on when the user was in a good mood were those that contained the keywords “free” and “deals,” and that many users were considering purchasing a product or service in the future.

On the other hand, the common denominator of advertisements that were more likely to be clicked on when users were in a bad mood was the content of the advertisements to relieve users of their complaints. It is very interesting to note that there were clear differences between the ad groups that were more likely to be clicked on in good and bad moods.

## Discussion

One of our future works is classification other affective states (beyond mood). We expect that the same framework can be used to model a wide range of affective states. Another task is to improve the model performance. In this study, we adopted a white-box model to examine the effectiveness of the model qualitatively. We aim at employing models focused on precision and recall performance for further performance improvement. We aim at discovering how the use of sensor and query data in different time periods affects the model performance of SMM and QMM.

The relationship between user mood, data obtained from the sensors, and query contents can change over time. In addition, there remains a question as to whether an estimation model constructed from the data of 460 participants could be applied to 11 million samples, despite confirming that the demographics of the 460 participants and the 11 million user sample were not significantly different. Increasing the number of user samples of the data that builds our model will be among our future works.

In conducting large-scale data collection experiments, issues such as the collection of highly private mobile data increased communication costs between client and server, and increased learning costs on the server where huge amounts of data are aggregated can be identified. In this study, learning on the client could consume significant computational resources of smartphones. Hence, all data were aggregated on the server.

As described in the Related Work section, there is a possibility that the above problem can be addressed by applying federal learning based on distributed machine learning to which a global model is learned by aggregating models that have been trained locally on data-generating clients. However, it is necessary to investigate the computational cost on the client that is acceptable to the subjects and the computational cost on the server as the data increase.

The data collection system and mood analysis engine constructed in this study can be regarded as a BD management system consisting of edge computing (EC) and cloud computing (CC). One of the future works is to introduce a more efficient and secure architecture to the BD management system constructed in this study. Examples include introducing more secure encryption methods for data transfers between EC and CC,^49,50^ and introducing algorithms to determine the optimal and energy-efficient allocation of resources to the BD management system.^51^

Also, building a secure network cache system could establish a more efficient architecture for large-scale data collection and utilization.^52^

The ethical issue is an important topic we need to be careful about. Since model building uses users' personal information, they may suffer from unintended information leakage. Smartphone sensors and search query data can be personally identifiable data, and distributed machine-learning methods and state-of-the-art secure communication and data management trends in the BD field should be implemented as indicated earlier.

If users do not agree to share information about their mood states, they may feel distrustful or insecure about having their affective states estimated as secondary data from their search queries. In addition, delivering advertisements based on users' mood states obtained through this research or applied to other services may be perceived by some users as an inappropriate use of their personal information, which raises ethical issues.

We must take steps to address these issues through legal regulations and guidelines regarding privacy and personal data protection. It is important that users are provided with clear information about how their data are collected and used (We believe that this paper will help). In addition, if data containing personal information are to be provided or shared with other services in a mood state, such as when data containing personal information are used to deliver advertisements, this must be clearly stated so that users can give their consent and self-determination.

## Conclusion

Affection awareness is one of the critical components of human-centric information services. However, in the real-world web field, estimating such states of the user is yet to be realized. Our research aims at estimating the mood states of web users from search queries, an easy-to-collect and non-invasive modality. Therefore, we built a search-QMM, which estimates the mood states of web users from search queries, an easy-to-collect and non-invasive modality.

Moreover, to boost the performance of the QMM, we proposed a novel two-step model building. For data augmentation, we built an SMM, which estimates from smartphone sensor data, and the output of the SMM is supplemented with ground-truth label data of the QMM. This two-step model building contributes to increasing label data of QMM and an 11% improvement in AUC. Our extensive user studies revealed multiple interesting results, including the changes in nationwide mood scores in multiple different time scale scenarios and the existence of the correlation between web advertisements and user mood.

One of the future works is applying the mood states of web users to the actual services through our deployed system. For example, when a web user has a negative mood state, the news web service can highlight some interesting news or present advertisements that can possibly cheer her up. We also aim at improving the acceptability of information by adaptively changing the notification method according to the mood states of the web user.

When the mood state of a web user is not normal, delaying the timing of notification from the system may increase the open notification rate. Although it should be emphasized that ethical issues must be addressed carefully, we believe that these case studies applying the estimated mood states have great potential to enrich our web-behavior.

## Abbreviations Used

AUC: area under the curve
BD: big data
CC: cloud computing
CCM: convergent cross-mapping
EC: edge computing
MM: multimodal
QMM: query mood model
SMM: sensor mood model
UM: unimodal

## Appendix

### Details of Modeling for Reproducibility

Improving reproducibility is one of the key factors ensuring our research's quality. Therefore, in this section, we explain the method to build SMM and QMM, and we describe the model performance in detail.

#### Detailed description of SMM building

In this study, we built the SMM, a model for estimating mood states from smartphone sensors to generate label data to be used as ground truth in building the QMM.

##### Data collecting

The SMM is built from smartphone sensor data and self-reported mood label data. As described in the Experiment for Building Models section, we conducted a 90-day data collection experiment with 460 participants. We installed our collection application based on the AWARE app^33,34^ on the participants' smartphones and collected eight types of smartphone sensor data (Table 1) and self-reported mood label data. The application allows us to set the collection sensor on/off and sensor frequency. It is also possible to set the destination for data sending, and the data collected in this experiment were sent to our server.

##### Data preprocessing

From the raw sensor data, we extracted a set of features in a 3-hour time window for each sensor. Table 4 summarizes the number of features extracted along with several representative feature types. The types of extracted features vary depending on the sensor type. The feature values to be calculated were determined by referring to several studies.^12,53^ Accelerometer and gyroscope sensors can collect the same types of values (score in the *x*, *y*, and *z*-axis), and we can extract the same type features based on the value of each sensor.

**Table 4.** Extracted features

***Sensor type***	***No. of features***	***Representative features***
Accelerometer, Gyroscope	23	(Mean, std, median, min, max) magnitude
		Mean (each axis)
		Variance (each axis)
		Skew (each axis)
		Kurtosis (each axis)
		Correlation (xy, yz, zx)
		Covariance (xy, yz, zx)
Barometer	5	(Mean, std, median, min, max) magnitude
Battery status	7	(Mean, std, median, min, max) battery level
		Number of charge times
		Duration of charge minutes
Location	12	Location entropy
		Number of location transitions
		Moving time percent
		Longest stay location information
		Second longest stay location information
		Third longest stay location information
Network type	5	Number of WiFi connectivity established
		Number of mobile connectivity established
		Most frequent network type
		Rate of WiFi
		Rate of mobile network
Weather (from OpenWeather)	50	Weather type
		(Mean, std, median, min, max) temperature
		(Mean, std, median, min, max) humidity
Screen status(on/off)	11	Number of unlocks (per minute)
		Number of interaction (per minute)

Regarding the label data, we treat the self-reported mood states as a three-class classification problem considering practical operability. The collected mood answers (originally on the seven-level Likert scale) were assigned to three different labels, −1 for “strongly negative,” “negative,” and “moderately negative,” 0 for “neutral,” and +1 for “strongly positive,” “positive,” and “moderately positive.”

We preprocess the dataset in two steps. First, we used multivariate imputation by the chained equations algorithm^54^ to impute missing values. Second, we used a standardization transformation to transform each variable into a standardized distribution.

##### Model building

We trained several linear and nonlinear classifier models, as shown in Table 5. We set the class_weight parameter of all ML models to “balanced” to deal with imbalanced labels and set other parameters to default values. To evaluate the model performance, we conducted a five-fold cross-validation. The data were randomly divided into 80% training data set and 20% test data set.

**Table 5.** Performance of different machine learning models

***Model***	***Micro-F1***	***Macro-F1***
Logistic Regression	0.370	0.331
Ridge Regression^56^	0.367	0.330
CART^57^	0.673	0.560
ExtraTree^58^	0.697	0.559
RandomForest^59^	0.719	**0.576**
XGBoost^60^	**0.747**	0.552
LightGBM^61^	0.662	0.559

Bold values signifies the better values.

We compared several machine-learning algorithms and used micro-*F*1 and macro-*F*1 scores, which are used in the evaluation of multi-class classification. Since the number of labels was unbalanced, we considered macro-*F*1 was important and chose random forest for the machine-learning algorithm, which revealed the best classification performance of macro-*F*1 score compared with others.

To improve the model performance, we optimized the random forest algorithm's hyperparameters using Optuna,^55^ a software framework for automating hyperparameter optimization. Table 6 summarizes the explored hyperparameter ranges, the number of trials, and the selected hyperparameters.

**Table 6.** Hyperparameter ranges, number of trials, and selected parameter

***Hyperparam***	***Range***	***Trials***	***Selected***
Criterion	[gini, ent]		gini
bootstrap	[true, false]		false
max_features	[auto, sqrt, log2]		sqrt
max_depth	[1, 1000]	100	880
max_leaf_nodes	[1, 1000]		981
min_samples_leaf	[1, 10]		10
min_samples_split	[2, 5]		3
n_estimators	[1, 1000]		953

##### Model performance

Table 7 shows the detailed results of the overall performance evaluation of the random forest after optimizing parameters. The score of macro-*F*1 increased from 0.576 to 0.597.

**Table 7.** Performance of sensor mood model

***Label***	***Precision***	***Recall***	***F1-score***
−1	0.40	0.36	0.37
0	0.59	0.61	0.60
1	0.82	0.82	0.82
micro avg	0.72	0.72	0.72
macro avg	0.60	0.59	0.60

We also calculated the feature importance of the SMM. The top-five feature types and importance scores were “mean-x-gyro” (0.053), “mean-z-gyro” (0.052), “mean-y-gyro” (0.051), “median-magnitude-acc” (0.037), and “median-magnitude-gyro” (0.025). We confirmed that many features with high importance scores were related to the gyroscope sensor.

#### Detailed description of QMM building

The QMM is a model that examines the relationship between the web search query and the user's mood score during the search behavior. After training, the user's mood score is classified from their search-query data.

##### Data collecting

The QMM is built from search queries and self-reported mood labels collected during a 90-day data collection experiment of 460 participants. Search query data, including timestamps, search keywords, and device information that is used for search, were sent to our managed server during the experiment. Device information is recorded as smartphone, tablet, or computer. As described in the Experiment for Building Models section, we informed the experiment participants that we were collecting these data.

Similar to the SMM, self-reported mood label data are collected through the application based on AWARE during the experiment. It notes that model training of both SMM and QMM used the same self-reported data that were collected during the experiment. As our proposed approach, label data of QMM training is a combination of the self-reported data collected during this experiment and the estimated outputs from the SMM for data imputation.

##### Data preprocessing

We set the length of the frame to 3 hours. All queries searched within the 3 hours were used as features. Because SMM-based mood scores are available 24 hours per day, the length of each frame can be shortened (fine-grained). However, if the length is too short, there is a risk that the questionnaire-based mood scores of the comparative method (QMM to be trained without the output from SMM; it is to be used in our comparative evaluation in Case Study: Nationwide Mood section) may become too sparse to be learned. Hence, the frames needed to be reasonably long. Therefore, we decided to use a frame length of 3 hours in this study.

We extracted two types of search query features of each three hourly frame: search query-derived features and search behavior-derived features. To create the search query-derived features, we first created a list of search words by dividing all search queries retrieved during the experiment by a space delimiter. We selected words from this list that at least 10 users retrieved.

Next, to construct the QMM to estimate mood states every 3 hours in this study, the search query history for each user is divided into three hourly segments. Search words were extracted from the 3-hour search queries by separating them with a space delimiter. We assigned 1 to each search word if it was included in the search word list created earlier and 0 if it was not. To create the search behavior-derived features, we extracted the number of searches, search devices (three types), and search timestamp.

As described in the Query Mood Model section, our key contribution is to increase the number of label data when training QMM by supplementing estimated outputs of the built SMM. Therefore, in this study, we prepared two types of label data for model evaluation: self-reported data only (without SMM) and adding the SMM outputs to self-reported data (with SMM). For practicality, we excluded the case where the value of ground truth is zero.

##### Model building

To investigate the validity of the trained QMMs qualitatively with the previously described features and label data (with SMM), QMM was trained with multiple model algorithms such as logistic regression, random forest, and xgboost. As with SMM, the class_weight parameter was set to “balanced,” and the other parameters were set to default values. To evaluate the model performance, we conducted a five-fold cross-validation.

It notes that the QMM is a binary classification model, so we calculated the *F*1 score and AUC for the model evaluation. Table 8 shows the result of each algorithm. Although the random forest performs better than other algorithms, we chose the logistic regression for the algorithm of the QMM since logistic regression has a lower training cost and a “white box” model with high model interpretability that is helpful in our real-world operation. The optimal value of the cost parameter c was determined by cross-validation ten times.

**Table 8.** Performance of different machine learning algorithm for query mood model (without sensor mood model)

***Model***	***F1***	***AUC***
Logistic Regression	0.552	0.567
RandomForest	0.591	0.579
XGBoost	0.533	0.531

##### Model performance

Table 9 shows the detailed results of the overall performance evaluation of QMM alone. The score of AUC increased from 0.567 to 0.583 after parameter tuning.

**Table 9.** Performance of query mood model (without sensor mood model)

***Label***	***Precision***	***Recall***	***F1-score***
−1	0.62	0.39	0.48
1	0.55	0.76	0.64
macro avg	0.59	0.57	0.57

Next, we compare two types of QMM (with SMM) and QMM (without SMM). Table 10 shows the number of data samples for positive (mood score is 1) and negative (mood score is −1) label data and AUC scores in the two types of QMM. We confirmed that the AUC increased from 0.583 to 0.623 (11%) in the case with additional data from SMM. The table also shows that the amount of training data more than doubled with SMM, indicating that the more than doubling of the dataset used for training by SMM contributed significantly to the improvement of model performance.

**Table 10.** Comparison of query mood model (with sensor mood model) and query mood model (without sensor mood model)

	***No. of positive data***	***No. of negative data***	***No. of label data***	***AUC***
QMM (with SMM)	24,500	1981	**26,481**	**0.623**
QMM (without SMM)	11,586	1545	13,131	0.583

Bold values signifies the better values.

The training data were balanced so that the amount of positive and negative data was the same before training by setting the class_weight parameter to “balanced,” which performed randomly undersampling the positive label data to match the negative label data. However, due to the imbalance in the training data, the amount of balanced label data decreased because of under-sampling. Moreover, the results of oversampling negative label data to match the positive label data according to the data distribution are shown in Table 11.

**Table 11.** Performance of query mood model by performing oversampling

	***No. before balancing***	***No. after balancing***	***AUC***
QMM (with SMM)	26,481	48,890	0.888
QMM (without SMM)	13,131	23,136	0.800

As with the down-sampling results, the amount of training data more than doubled by SMM, and the AUC of our proposed model increased from 0.800 to 0.888 compared with our baseline QMM without SMM.

##### Query features constructed for QMM

When we investigated the trained QMM, we qualitatively confirmed that the model represents the mood. Among all the search queries collected during the experiment, 81,000 queries that were searched two or more times in the experiment were trained since the query that was searched only once cannot be a feature. The number of queries weighted in the trained model (out of all 81,000 query words) was 217. Table 12 shows examples of representative queries that had top-five high weights in the trained QMM. (Note that all descriptions in this table were translated into English; the original queries are in Japanese. The record with $ is the explanation of the query content rather than the actual content, and this is to protect the business and website.)

**Table 12.** Top-five query features with high weight

***Positive queries***	***Negative queries***
$shopping comparison website$	“I want to die”
“baby”	“pachinko” (Japanese pinball gamble)
$Smartphone walk-around game$	“credit card”
$celebrity blog website$	“temporary worker”
$gourmet comparison website$	“headache”
$website for checking utility usage/price$	$fraudulent site$

For example, the top positive words include the names of shopping (price) comparison websites for home appliances and restaurants and the name of a smartphone game for walking around outside, indicating the importance of users actively obtaining a variety of information. Moreover, direct expressions such as “pachinko” (Japanese-style pinball gambling) and “I want to die” were ranked as negative words, not to mention that they fit our intuition.
