# Supplementary material for: Large-Scale Estimation and Analysis of Web Users' Mood from Web Search Query and Mobile Sensor Data
Source: Big Data. 2024 Jun 19;12(3):191–209. doi: 10.1089/big.2022.0211 (PMC11304759; doi:10.1089/big.2022.0211)
Supplement: Supplementary Material [file big.2022.0211_suppl_material.docx]

### A.1 Detailed Description of SMM Building

In this study, we built the SMM, a model for estimating mood states from smartphone sensors to generate label data to be used as ground truth in building the QMM.

#### A.1.1 Data Collecting

SMM is built from smartphone sensor data and self-reported mood label data. As described in Section 3, we conducted a 90-day data collection experiment with 460 participants. We installed our collection application based on the AWARE app (17; 36) on the participants’ smartphones and collected eight types of smartphone sensor data (table 1) and self-reported mood label data. The application allows us to set the collection sensor on/off and sensor frequency. It is also possible to set the destination for data sending, and the data collected in this experiment was sent to our server.

#### A.1.2 Data Preprocessing

From the raw sensor data, we extracted a set of features in a three-hour time window for each sensor. Table 4 summarizes the number of features extracted along with several representative feature types. The types of extracted features vary depending on the sensor type. The feature values to be calculated were determined by referring to several studies (29; 5). Accelerometer and gyroscope sensors can collect the same types of values (score in the x, y, and z-axis), and we can extract the same type features based on the value of each sensor.

Regarding the label data, we treat the self-reported mood states as a three-class classification problem considering practical operability. The collected mood answers (originally on the seven-level Likert scale) were assigned to three different labels, $-1$ for “strongly negative,” “negative,” and “moderately negative,” $0$ for “neutral,” and $+1$ for “strongly positive,” “positive” and “moderately positive.”

We preprocess the dataset in two steps. First, we used multivariate imputation by chained equations (MICE) algorithm (47) to impute missing values. Second, we used a standardization transformation to transform each variable into a standardized distribution.

#### A.1.3 Model Building

We trained several linear and nonlinear classifier models, as shown in Table 5. We set the class_weight parameter of all ML models to “balanced” to deal with imbalanced labels and set other parameters to default values. To evaluate the model performance, we conducted a five-fold cross-validation. The data were randomly divided into 80% training data set and 20% test data set.

We compared several machine-learning algorithms and used micro-F1 and macro-F1 scores, which are used in the evaluation of multi-class classification. Since the number of labels was unbalanced, we considered macro-F1 was important and chose random forest for the machine-learning algorithm, which revealed the best classification performance of macro-F1 score compared with others.

To improve the model performance, we optimized the random forest algorithm’s hyperparameters using Optuna (4), a software framework for automating hyperparameter optimization. Table 6 summarizes the explored hyperparameter ranges, the number of trials, and the selected hyperparameters.

#### A.1.4 Model Performance

Table 7 shows the detailed results of the overall performance evaluation of the random forest after optimizing parameters. The score of macro-F1 increased from 0.576 to 0.597.

We also calculated the feature importance of the SMM. The top-five feature types and importance scores were “mean-x-gyro”(0.053), “mean-z-gyro” (0.052), “mean-y-gyro” (0.051), “median-magnitude-acc” (0.037) and “median-magnitude-gyro” (0.025). We confirmed that many features with high importance scores were related to the gyroscope sensor.

### A.2 Detailed Description of QMM Building

QMM is a model that examines the relationship between the web search query and the user’s mood score during the search behavior. After training, the user’s mood score is classified from their search-query data.

#### A.2.1 Data Collecting

QMM is built from search queries and self-reported mood labels collected during a 90-day data collection experiment of 460 participants. Search query data including timestamps, search keywords, and device information that is used for search, were sent to our managed server during the experiment. Device information is recorded as smartphone, tablet, or computer. As described in Section 3, we informed the experiment participants that we were collecting these data. Similar to the SMM, self-reported mood label data are collected through the application based on AWARE during the experiment. It notes that model training of both SMM and QMM used the same self-reported data which were collected during the experiment. As our proposed approach, label data of QMM training is a combination of the self-reported data collected during this experiment and the estimated outputs from the SMM for data imputation.

#### A.2.2 Data Preprocessing

We set the length of the frame to three hours. All queries searched within the three hours were used as features. Because SMM-based mood scores are available 24 h per day, the length of each frame can be shortened (fine-grained). However, if the length is too short, there is a risk that the questionnaire-based mood scores of the comparative method (QMM to be trained without the output from SMM; it is to be used in our comparative evaluation in Section 6) may become too sparse to be learned. Hence, the frames needed to be reasonably long. Therefore, we decided to use a frame length of three hours in this study.

We extracted two types of search query features of each three hourly frame: search query-derived features and search behavior-derived features. To create the search query-derived features, we first created a list of search words by dividing all search queries retrieved during the experiment by a space delimiter. We selected words from this list that at least ten users retrieved. Next, to construct the QMM to estimate mood states every three hours in this study, the search query history for each user is divided into three hourly segments. Search words were extracted from the three-hour search queries by separating them with a space delimiter. We assigned 1 to each search word if it was included in the search word list created earlier and 0 if it was not. To create the search behavior-derived features, we extracted the number of searches, search devices (three types), and search timestamp.

As described in Section 4.3, our key contribution is to increase the number of label data when training QMM by supplementing estimated outputs of the built SMM. Therefore, in this study, we prepared two types of label data for model evaluation: self-reported data only (without SMM) and adding the SMM outputs to self-reported data (with SMM). For practicality, we excluded the case where the value of ground truth is zero.

**A.2.3** **Model Building**

To investigate the validity of the trained QMMs qualitatively with the previously described features and label data (with SMM), QMM was trained with multiple model algorithms such as logistic regression, random forest, and xgboost. As with SMM, the class_weight parameter was set to “balanced,” and the other parameters were set to default values. To evaluate the model performance, we conducted a five-fold cross-validation. It notes that the QMM is a binary classification model, so we calculated the F1 score and AUC for the model evaluation. Table 8 shows the result of each algorithm. Although the random forest performs better than other algorithms, we chose the logistic regression for the algorithm of the QMM since logistic regression has a lower training cost and a “white box” model with high model interpretability which is helpful in our real-world operation. The optimal value of the cost parameter c was determined by cross-validation ten times.

#### A.2.4 Model Performance

Table 9 shows the detailed results of the overall performance evaluation of QMM alone. The score of AUC increased from 0.567 to 0.583 after parameter tuning.

Next, we compare two types of QMM (with SMM) and QMM (without SMM). Table 10 shows the number of data samples for positive (mood score is 1) and negative (mood score is -1) label data and AUC scores in the two types of QMM. We confirmed that the AUC increased from 0.583 to 0.623 (11%) in the case with additional data from SMM. The table also shows that the amount of training data more than doubled with SMM, indicating that the more than doubling of the dataset used for training by SMM contributed significantly to the improvement of model performance.

The training data were balanced so that the amount of positive and negative data was the same before training by setting the class_weight parameter to “balanced,” which performed randomly undersampling the positive label data to match the negative label data. However, due to the imbalance in the training data, the amount of balanced label data decreased because of under-sampling. Moreover, the results of oversampling negative label data to match the positive label data according to the data distribution are shown in Table 11. As with the down-sampling results, the amount of training data more than doubled by SMM, and the AUC of our proposed model increased from 0.800 to 0.888 compared with our baseline QMM without SMM.

#### A.2.5 Query Features Constructed for QMM

When we investigated the trained QMM, we qualitatively confirmed that the model represents the mood. Among all the search queries collected during the experiment, 81,000 queries that were searched two or more times in the experiment were trained since the query which was searched only once cannot be a feature. The number of queries weighted in the trained model (out of all 81,000 query words) was 217. Table 12 shows examples of representative queries that had top-five high weights in the trained QMM. (Note that all descriptions in this table were translated into English; the original queries are in Japanese. The record with $ is the explanation of the query content rather than the actual content, and this is to protect the business and website.) For example, the top positive words include the names of shopping (price) comparison websites for home appliances and restaurants and the name of a smartphone game for walking around outside, indicating the importance of users actively obtaining a variety of information. Moreover, direct expressions such as “pachinko” (Japanese-style pinball gambling) and “I want to die” were ranked as negative words, not to mention that they fit our intuition.
